# The Role of Cytokines, Chemokines, and Growth Factors in the Pathogenesis of Pityriasis Rosea

**DOI:** 10.1155/2015/438963

**Published:** 2015-09-10

**Authors:** Francesco Drago, Giulia Ciccarese, Francesco Broccolo, Massimo Ghio, Paola Contini, Hajdhica Thanasi, Aurora Parodi

**Affiliations:** ^1^Section of Dermatology, IRCCS Azienda Ospedaliera Universitaria San Martino-IST, 16132 Genoa, Italy; ^2^Laboratory of Microbiology and Virology, Department of Health Sciences, Faculty of Medicine and Surgery, University of Milano-Bicocca, Via Cadore, 48, 20900 Monza, Italy; ^3^Department of Internal Medicine, IRCCS Azienda Ospedaliera Universitaria San Martino-IST and University of Genoa, 16132 Genoa, Italy

## Abstract

*Introduction*. Pityriasis rosea (PR) is an exanthematous disease related to human herpesvirus- (HHV-) 6/7 reactivation. The network of mediators involved in recruiting the infiltrating inflammatory cells has never been studied. *Object*. To investigate the levels of serum cytokines, growth factors, and chemokines in PR and healthy controls in order to elucidate the PR pathogenesis. *Materials and Methods*. Interleukin- (IL-) 1, IL-6, IL-17, interferon- (IFN-) *γ*, tumor necrosis factor- (TNF-) *α*, vascular endothelial growth factor (VEGF), granulocyte colony stimulating factor (G-CSF), and chemokines, CXCL8 (IL-8) and CXCL10 (IP-10), were measured simultaneously by a multiplex assay in early acute PR patients' sera and healthy controls. Subsequently, sera from PR patients were analysed at 3 different times (0, 15, and 30 days). *Results and discussion*. Serum levels of IL-17, IFN-*γ*, VEGF, and IP-10 resulted to be upregulated in PR patients compared to controls. IL-17 has a key role in host defense against pathogens stimulating the release of proinflammatory cytokines/chemokines. IFN-*γ* has a direct antiviral activity promoting NK cells and virus specific T cells cytotoxicity. VEGF stimulates vasculogenesis and angiogenesis. IP-10 can induce chemotaxis, apoptosis, cell growth, and angiogenesis. *Conclusions*. Our findings suggest that these inflammatory mediators may modulate PR pathogenesis in synergistic manner.

## 1. Introduction

Pityriasis rosea (PR) is an exanthematous disease associated with the systemic reactivation of human herpesvirus- (HHV-) 6 and/or 7 [1–6]. It usually begins with a single, erythematous plaque (herald patch) followed in about 2 weeks by smaller lesions on the cleavage lines of the trunk (Christmas tree distribution). The duration may vary but usually it gradually disappears in 4 weeks. Up to 69% of the patients experience prodromal symptoms as fever, headache, arthralgia, or malaise [5]. The constitutional symptoms, the frequent clustering of cases, and the almost complete absence of recurrent episodes support the infectious etiology of the disease [5]. The large positivity of HHV-6/7 in general population, the low human-to-human transmission rate, the variable severity of the eruption, its occurrence, and recurrences in states of altered immunity all favor the hypothesis that PR is a clinical presentation of HHV-6 and/or HHV-7 reactivation [1–6]. Immunohistological studies showed the presence of T cells and Langerhans cells within the inflammatory dermal infiltrate of PR lesions, suggesting a role of the cell-mediated immunity in the disease [7]. We previously demonstrated that fractalkine, the only member of the *δ*-chemokines, and interleukin- (IL-) 22, a cytokine expressed by Th17 cells, are increased in sera of active PR patients [8, 9]. The involvement of these chemokines, which promote the antimicrobial defense and protect against damage, suggests an active immunological response in PR. However, the knowledge on the PR pathogenesis and its cytokine profile is still poorly understood.

To elucidate the mechanisms that underlie PR, the levels of serum inflammatory cytokines, growth factors, and chemokines were investigated in the same samples from PR patients and healthy controls correlating them with PR activity.

## 2. Subjects and Methods

### 2.1. Patients and Controls

The study included 24 patients (10 males and 14 females, mean age: 27.5 ± 9.5 years, age range: 7–49 years) with PR. The inclusion criteria were patients who sought care for a skin rash at our Dermatology Department between January and June 2014 who gave informed consent to take blood samples for laboratory investigations and in whom a final diagnosis of PR was made. All patients had the classical clinical findings of PR and the diagnosis was made clinically. Twenty-four healthy blood donors, sex- and age-matched (11 males and 13 females, mean age: 29.0 ± 8.0 years, age range: 9–51 years), were enrolled as control. Each subject gave a written informed consent to the study. Exclusion criteria were (a) all PR patients who have received cytostatic or immunosuppressive drugs and (b) all PR patients with atypical rash (or with infectious diseases).

### 2.2. Serum Samples

Blood samples from PR patients were collected at different time points:

- onset of the disease, within 3 weeks from the beginning of the eruption, when usually the herald patch and some smaller lesions just appear (time 0);
- postacute phase (after 15 days) that usually corresponds to the peak of the clinical manifestations (time 1);
- convalescent phase or remission (28 days from the beginning of the eruption) when the clinical resolution is often obtained (time 2).

Blood was collected in endotoxin-free silicone-coated tubes without additive. The blood samples were allowed to clot at room temperature for 30 minutes before centrifugation (1000 ×g, 15 minutes); the serum was removed and stored at −80°C until analysed.

### 2.3. Assessment of Serum Cytokines Levels by a Multiplex Assay

Serum levels of cytokines and chemokines including IL-1, IL-6, IL-17, interferon- (IFN-) *γ*, tumor necrosis factor- (TNF-) *α*, vascular endothelial growth factor (VEGF), granulocyte colony stimulating factor (G-CSF), and chemokines, CXCL8 (IL-8) and CXCL10 (IP-10), were measured by a multiplex cytokine array system (Luminex Inc., Austin, TX, USA) as previously described [10].

### 2.4. Statistical Analyses

Statistical analyses were performed using Prism 6 software. Continuous variables were presented as mean ± standard error of the mean (SEM), whereas categorical variables were presented as absolute and relative frequencies. Mann-Whitney's test was used to compare continuous data between the studied and control groups. Pearson's correlation coefficient was used in correlation analyses and 0.05 significant level was assumed in statistical tests.

## 3. Results 

### 3.1. Diverse Cytokine Profiles of PR Patients and Healthy Controls

#### 3.1.1. Cross-Sectional Evaluation

To facilitate functional interpretation of results, cytokines were sorted into three functional groups: a group denoted “cellular cytokines” which drive, albeit not exclusively, cytotoxic and antiviral responses (e.g., IL-1, IL-6, IL-17, TNF-*α*, and IFN-*γ*), a group denoted “growth and angiogenic factors” (e.g., VEGF and G-CSF), and a group denoted “chemokines” (IL-8 and IP-10). In each group, we compared serum cytokines levels in patients with PR and controls.

#### 3.1.2. Evaluation of Selected Serum Cytokines Levels in PR Patients: A Cross-Sectional Evaluation

**Figure 1:**
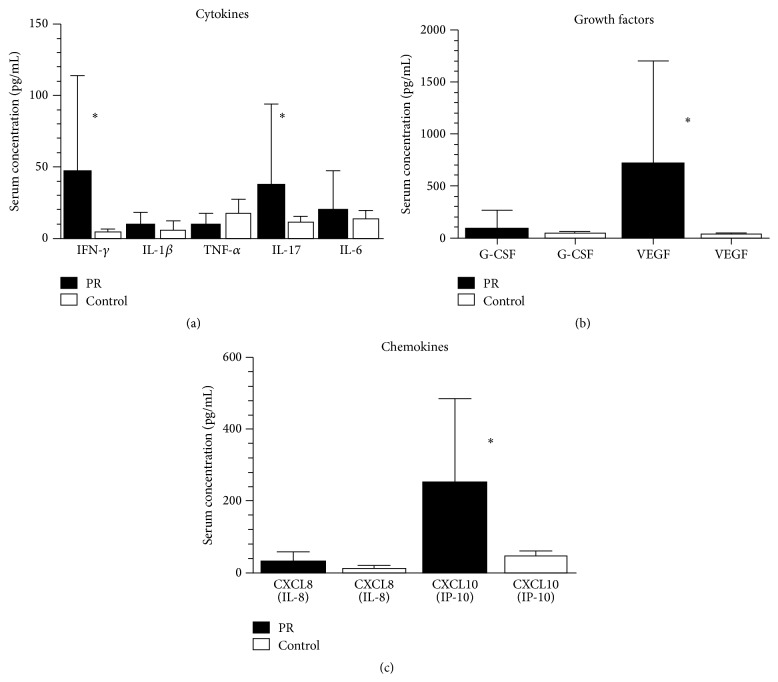
Comparison of various serum cytokine levels (circulating cellular cytokines, growth factors, and chemokines) between PR patients (*n* = 24) and controls (*n* = 24). Bars show the mean in pg/mL and SEM. ^*∗*^Significantly different from compared values by Mann-Whitney *U* test (*P* < 0.005).

Figure 1 summarizes the serum cellular cytokines, chemokines, and growth and angiogenic factors levels of patients and controls at the onset of the disease.

Among the “cellular cytokines,” only IFN-*γ* (*P* < 0.0001) and IL-17 (*P* = 0.0008) were significantly increased in PR patients compared with the levels found in healthy individuals (Figure 1(a)). The comparison of the serum levels of growth factors revealed a significant increase of VEGF (*P* < 0.0001) in PR patients compared to healthy controls. Among the chemokines, a marked increase of CXCL10 (*P* < 0.0056) in PR patients was detected compared to controls.

#### 3.1.3. Evaluation of Selected Serum Cytokines Levels in PR Patients: A Longitudinal Analysis

No significant difference was found among cytokines, chemokines, and growth factors levels at the different time points, suggesting that there was no correlation between cytokines levels and disease activity in PR.

**Figure 2:**
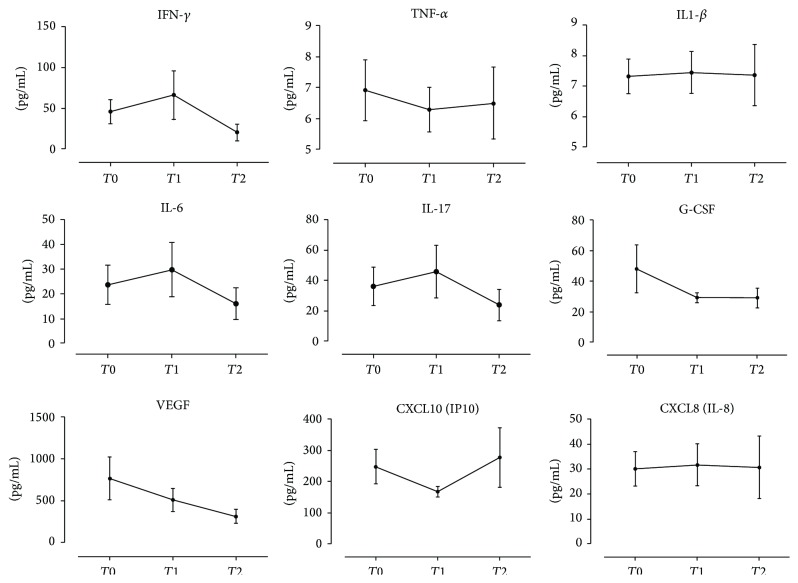
Cytokine levels at different time points in PR patients: at onset of the disease (*T*_0_), after 15 days (*T*_1_), and after 30 days (*T*_2_).

Figure 2 shows the cellular cytokines levels and the chemokines and growth factors levels at the different time point in PR patients: no significant changes were detected, except for a decrease of VEGF from the onset until clinical resolution.

#### 3.1.4. Differences in Cytokines Levels in Onset PR Patients

Spearman's rank correlation was used for calculating the correlations between the different cytokines (evaluating all possible combinations of cytokines levels) in onset PR patients. We performed correlation analysis among all studied cytokines. We found an intrasubject correlation between IFN-*γ* and IL-17 (*r* = 0.49 and *P* = 0.008), IFN-*γ* and VEGF (*r* = 0.61 and *P* = 0.005), and IFN-*γ* and IP-10 (*r* = 0.54 and *P* = 0.006). A positive correlation was also found between IL-17 and VEGF (*r* = 0.58 and *P* = 0.009), IL-17 and IP-10 (*r* = 0.51 and *P* = 0.003), and VEGF and IP-10 (*r* = 0.55 and *P* = 0.004). Finally, we did not find any correlation between other pairs of studied cytokines.

## 4. Discussion

HHV-6 and HHV-7 are closely related viruses, members of the* Roseolovirus* genus of the HHVs, largely diffuse in the general population (seroprevalence in the healthy adult population is 80–90%), and commonly acquired in early childhood. Saliva, through which HHV-6 is chronically shed, is probably the usual mode of transmission. These viruses are able to cause a primary infection that is usually asymptomatic or may cause the exanthema subitum or a febrile illness without any rash rarely accompanied by convulsions after which they establish a latent infection. HHV-6 persists in salivary glands and possibly also in terminal bronchi and neuroglial cells. HHV-7 persists in CD4 T lymphocytes and also in the epithelia of salivary glands, bronchi, and cells of the skin, liver, and kidney [5].

During all HHVs infections, the cell mediated immunity is crucial for the control of the viral infection and replication. The latency is maintained particularly by the infiltrating CD4 and CD8 T cells in the skin or mucosa and in the dorsal root ganglion where the replication takes place. Those lymphocytes are specific for the structural proteins of the virus, lie in apposition to neurons, and secrete IFN-*γ*. These viruses may reactivate possibly due to a drop in the cell-mediated surveillance in occasion of other infections, exposure to endotoxins, or endocrine stimulation (including stress situations) [5].

The relationship between PR and HHV-6 and HHV-7 has been well established [1–5] but the elusive pathogenesis of the disease remains a subject of interest. We have shown previously that HHV-7 or HHV-6 is active during the early stage of PR, suggesting that they might play an etiological role in this disease. In addition, we have shown that plasma load of HHV-6 and HHV-7, a direct marker of viral replication, is associated with the development of systemic symptoms as well as with a significant reduction of the humoral neutralizing response against HHV-7, further suggesting that PR may be because of the endogenous reactivation of HHV-7 or HHV-6 infection [6].

Moreover, within the dermal infiltrate of PR lesions, an increase in Langerhans cells and presence of activated T cells (with an increased CD4+ versus CD8+ T ratio) have been described, confirming that the interaction between T-helper, Langerhans cells, and inflammatory dendritic dermal and epidermal cells (IDECs) may represent a pathogenetic mechanism in PR in a virus-triggered microenvironment [7]. In fact, CD4+ T cells, together with the salivary glands, are sites where the HHV-6 and HHV-7 infection state is established [11, 12].

Studies of the cytokine profile in PR are necessary to decipher the role of the activated T cells and to further characterize the T-helper (Th1 and Th2) paradigm in PR.

Recent studies challenged the Th1/Th2 paradigm by discovering several T-helper cell subsets with specific differentiation program and functions, including Th17 cells, regulatory T (Treg) cells, and follicular helper T (Tfh) cells. Th17 cells are characterized by production of IL-17, IL-21, IL-22, and other cytokines. IL-21, besides stimulating the proliferation and differentiation of activated leukocytes, acts also by an autocrine mechanism on Th17 cells, stimulating in turn the production of IL-17 and the other cytokines [13].

Our study is the first on the cytokine network in PR. The key cytokines that discriminate between onset PR and healthy individuals were IFN-*γ*, IL-17, VEGF, and CXCL10.

We speculate the putative role of these key cytokines in PR pathogenesis.

### 4.1. IL-17

The serum IL-17 levels were found to be significantly higher than healthy controls (*P* < 0.001). An intrasubject positive correlation was also found between IL-17 and VEGF (*r* =  0.58 and *P* = 0.009) and between IL-17 and IP-10 (*r* = 0.51 and *P* = 0.003). Similar to other authors, we have also found a positive correlation between IL-17 and VEGF levels confirming the involvement of IL-17 in angiogenesis [14–16]. To the best of our knowledge, a positive correlation between the other pairs of cytokines assessed in our study has never been described.

IL-17 has a key role in host defense against certain pathogens through stimulating the release of antimicrobial peptides and proinflammatory cytokines and chemokines [13, 17].

The significant increase of IL-17 serum levels in PR patients indirectly supports the involvement of HHV-6/HHV-7 in the PR pathogenesis, and it has been demonstrated for IL-22, another Th17 cytokine [9]. In fact, an antigenic trigger, as a reactivated quiescent HHV-6/HHV-7, via the Th17 cells stimulation and the IL-21 production, could boost IL-22 and IL-17 secretion by an autocrine mechanism [9, 18, 19]. IL-17, enhancing the production of proinflammatory and antimicrobial molecules, could start an inflammatory response that limits the spread of the HHV-6/HHV-7 reactivation. In fact, PR has a self-limited behavior lasting about 4–8 weeks.

### 4.2. IFN-*γ*

IFN-*γ*, produced mainly by T cells and NK cells, has a direct antiviral activity promoting NK cells and virus specific T cells cytotoxicity. In fact, it is considered an important molecule involved in antiviral host defense. Acting via its receptor, IFN-*γ* activates hundreds of genes leading to proinflammatory effects by increasing antigen processing and presentation and anti-inflammatory effects due to its apoptotic and antiproliferative functions. In our study, we demonstrated an intrasubject positive correlation between IFN-*γ* and IL-17 (*r* = 0.49 and *P* = 0.008), IFN-*γ* and VEGF (*r* = 0.61 and *P* = 0.005), and IFN-*γ* and IP-10 (*r* = 0.54 and *P* = 0.006).

Usually, acute viral infections induce an increase in the serum levels of IFN-*γ* [20]. In fact, this cytokine has been demonstrated to be the most sensitive marker of the CD4+ T cells response to the HHV-6 acute infection [21]. In our study, we found IFN-*γ* plasma levels to be significantly higher in PR patients compared to controls, especially in the samples collected after 15 days from diagnosis, which usually corresponds to the peak of the clinical manifestations (time 1).

Conversely, one important study found a significant decrease in the serum level of IFN-*γ* in patients with PR compared to healthy controls and postulated that such decrease is linked to the decreased number or impaired function of peripheral CD4+ T cells in PR patients [22]. Unfortunately, in that study, it was not specified in which phase of the eruption the blood samples were collected. We can hypothesize that it was done during a very early stage of the disease when a transiently weakened Th1 response to the latent HHV-6/HHV-7 infection with a temporary lower expression of IFN-*γ* may occur.

### 4.3. VEGF

Among the 5 VEGFs, VEGF-A is the most potent vascular permeability agent. It is produced by various cell types (including endothelial cells) and stimulates vasculogenesis and angiogenesis following its binding with tyrosine kinase receptors (VEGFRs) [23]. In the herpetic stromal keratitis, it has been recently demonstrated that HSV-1 infected corneal epithelial cells are the primary source of VEGF-A during acute ocular infection [24]. Moreover, in many inflammatory skin diseases associated with vascular hyperpermeability (atopic dermatitis, psoriasis, and dermatitis herpetiformis), an overproduction of VEGF in lesional keratinocytes has been demonstrated. Although the mechanism is still unclear, it is possible that keratinocytes might release greater amounts of VEGF which in turn could contribute to the subsequent increase in plasma concentration. Analogous cellular types may be responsible for VEGF increase in PR patients: not only lesional keratinocytes but also HHV-6/HHV-7 infected peripheral blood mononuclear cells (PBMCs) may be able to synthesize and release VEGF during all the phases of the exanthem. Whether circulating VEGF contributes directly or indirectly to PR pathogenesis or it is merely a secondary phenomenon needs to be determined. In our study, we demonstrated an intrasubject positive correlation between VEGF and IP-10 (*r* = 0.55 and *P* = 0.004).

Notable, viral pattern recognition receptors almost universally activate IFN pathways [20] and IFN, especially type I (IFN-*α* and IFN-*β*), inhibits expression of VEGF-A [25]. In fact, in our PR patients, the blood interferon increase at *T*_1_ (usually the peak of the clinical manifestations) corresponds to a VEGF decrease at the same time point.

### 4.4. CXCL10/IP-10

IP-10 is an IFN-inducible chemokine secreted by many cell types (neutrophils, eosinophils, monocytes, epithelial cells, endothelial cells, stromal cells, and keratinocytes) that can induce chemotaxis, apoptosis, cell growth, and angiogenesis.

It plays a major role in the control of viral replication as it is highly upregulated by type I and type II IFN and strongly promotes the chemotaxis of NK, CD4+, and CD8+ T cells [26–28].

Our study suggests that CXCL10 is upregulated by IFN-*γ*, since an intrasubject positive correlation between IFN-*γ* and IP-10 (*r* = 0.54 and *P* = 0.006) was found and both mediators resulted to be significantly increased in PR patients compared to controls. CXCL10 levels rose mainly during the final phase of the disease (*T*_2_), whereas IFN-*γ* has a major increment during the PR “intermediate” phase, corresponding to the peak of the clinical manifestation of the exanthem. Therefore, it may be hypothesized that IFN-*γ* upregulates CXCL10 with a certain latency.

## 5. Conclusions

In conclusion, our study investigated the cytokines and chemokines network in PR, providing evidence that circulating IL-17, IFN-*γ*, VEGF, and CXCL10 are increased in PR patients. The present results underscore the active immunological response in PR and may contribute to a better definition of the skin defense network. Incidentally, the cytokine pattern could support a viral induced disease process in PR pathogenesis.
